# Interleukin-1 participates in the classical and alternative activation of microglia/macrophages after spinal cord injury

**DOI:** 10.1186/1742-2094-9-65

**Published:** 2012-04-07

**Authors:** Atsushi Sato, Hirokazu Ohtaki, Tomomi Tsumuraya, Dandan Song, Kenji Ohara, Masahide Asano, Yoichiro Iwakura, Takashi Atsumi, Seiji Shioda

**Affiliations:** 1 Department of Anatomy, Showa University School of Medicine, 15-8 Hatanodai, Shinagawa-ku, Tokyo 142-8555, Japan; 2 Department of Orthopedic Surgery, Showa University Fujigaoka Hospital, 1-30 Fujigaoka, Aoba-ku, Yokohama, Kanagawa 227-8501, Japan; 3 Division of Transgenic Animal Science, Advanced Science Research Center, Kanazawa University, Kanazawa, Ishikawa 920-1192, Japan; 4 The Institute of Medical Science, Laboratory of Animal Research Center, University of Tokyo, 4-6-1 Shirokanedai, Minato-ku, Tokyo 108-8639, Japan

**Keywords:** Spinal cord injury, Microglial cells, Interleukin-1, Interleukin-4, Mice

## Abstract

**Background:**

Microglia and macrophages (MG/MΦ) have a diverse range of functions depending on unique cytokine stimuli, and contribute to neural cell death, repair, and remodeling during central nervous system diseases. While IL-1 has been shown to exacerbate inflammation, it has also been recognized to enhance neuroregeneration. We determined the activating phenotype of MG/MΦ and the impact of IL-1 in an *in vivo *spinal cord injury (SCI) model of IL-1 knock-out (KO) mice. Moreover, we demonstrated the contribution of IL-1 to both the classical and alternative activation of MG *in vitro *using an adult MG primary culture.

**Methods:**

SCI was induced by transection of the spinal cord between the T9 and T10 vertebra in wild-type and IL-1 KO mice. Locomotor activity was monitored and lesion size was determined for 14 days. TNFα and Ym1 levels were monitored to determine the MG/MΦ activating phenotype. Primary cultures of MG were produced from adult mice, and were exposed to IFNγ or IL-4 with and without IL-1β. Moreover, cultures were exposed to IL-4 and/or IL-13 in the presence and absence of IL-1β.

**Results:**

The locomotor activity and lesion area of IL-1 KO mice improved significantly after SCI compared with wild-type mice. TNFα production was significantly suppressed in IL-1 KO mice. Also, Ym1, an alternative activating MG/MΦ marker, did not increase in IL-1 KO mice, suggesting that IL-1 contributes to both the classical and alternative activation of MG/MΦ. We treated primary MG cultures with IFNγ or IL-4 in the presence and absence of IL-1β. Increased nitric oxide and TNFα was present in the culture media and increased inducible NO synthase was detected in cell suspensions following co-treatment with IFNγ and IL-1β. Expression of the alternative activation markers Ym1 and arginase-1 was increased after exposure to IL-4 and further increased after co-treatment with IL-4 and IL-1β. The phenotype was not observed after exposure of cells to IL-13.

**Conclusions:**

We demonstrate here in *in vivo *experiments that IL-1 suppressed SCI in a process mediated by the reduction of inflammatory responses. Moreover, we suggest that IL-1 participates in both the classical and alternative activation of MG in *in vivo *and *in vitro *systems.

## Introduction

Every year more than 10,000 people in the United States are victims of spinal cord injury caused by traffic, sports and other accidents. While medication during the acute injury period involves the administration of large doses of steroid and other anti-inflammatory drugs, the recovery of neurological functions relies on the host's neural plasticity and compensatory mechanisms. Many of these patients are permanently paralyzed [1,2]. The spinal cord injury (SCI) site increases initially due to the invasion of monocytes such as macrophages (MΦ) and microglial cells (MG) which are resident macrophages in neural tissue. MΦ and MG (MΦ/MG) activation increases neuroinflammation by releasing pro-inflammatory cytokines such as IL-1β and TNFα, as well as reactive oxygen species such as superoxide anion and nitric oxide (NO). This inflammatory spiral gives rise to astroglial scar formation around the injury epicenter and inhibits the tissue repair process and neuroregeneration [2].

MΦ (and perhaps MG as well) have a diverse range of functions during inflammatory periods depending on the type of induction caused by unique cytokine stimuli [3-5]. The classically activated MΦ brought about by IFNγ, induces the production of IL-1β, TNFα and NO from inducible NO synthase (iNOS), and works as a cytotoxic phenotype in which central nervous system (CNS) damage is exacerbated by inflammation. In contrast, it is considered that alternatively activated MΦ, which are also known as type M2 MΦ and are induced by the stimulation of IL-4 and IL-13, may be involved in tissue repair and remodeling [5-7]. MΦ activated in this manner promote axonal growth and overcome inhibitory substrates [8]. Such MΦ implanted into the injured spinal cord have been reported to induce an increase in axonal regrowth or functional improvement [9,10]. We have also reported that transplanted human stem/progenitor cells from bone marrow (hMSCs) rescued neural cell death in the hippocampus after global ischemia; this process is mediated by the induction of the alternatively activated MΦ/MG which is reflected by expression of the marker Ym1 [11]. Like these, although MΦ/MG might tune tissue damage to repair after CNS injuries, there is little evidence to illustrate the phenotypes after the injuries. Therefore, we demonstrate the MΦ/MG activating phenotype after SCI.

IL-1 plays a crucial role in CNS damage [12-14]. IL-1 contributes to an increase in the size of the lesion after mechanical- and chemical-induced SCI, while treatment with an IL-1 receptor antagonist (IL-1ra) reduced this effect [13-16]. Also in the stroke model, treatment with IL-1β has exacerbated ischemic brain damage [17] while IL-1ra [18] or IL-1 gene-deficient (KO) mice have decreased infarct volumes [19-21]. One of the functions of IL-1 is activation of MG/MΦ, and IL-1 and its receptors expressed by MG/MΦ regulate NO synthesis, apoptosis and secondary inflammatory responses [13,19,21,22]. Recent evidence suggests that IL-1β triggers the proliferation and early differentiation of neural progenitor cells during the development of the spinal cord and after hippocampal injury [23,24]. It also activates type II helper T cell induction which has an anti-inflammatory effect after cerebral ischemia [25]. Moreover, other inflammatory factors, such as TNFα and iNOS, have been implicated in the neural regeneration process during tissue repair [26,27]. Therefore, IL-1 or inflammatory factors could play roles in tissue repair during subacute periods. Although the alternative activating phenotype of MΦ/MG has been suggested in neural repair processes and IL-1 might also contribute to neuroregeneration, the participation of IL-1 against the alternative activation of MΦ/MG has not been reported yet.

In the present study, we compared lesion size after SCI between IL-1 KO and wild-type mice. We then determined MΦ/MG activation by measuring marker levels for 14 days after SCI.

Moreover, we determine that IL-1 influences MG directly to modulate the alternative activation process in adult mouse primary MG cells obtained from IL-1 KO and wild-type mice.

## Materials and methods

### Animals

Mice with homozygous disruption of both IL-1α and β genes (IL-1 KO) have been described previously [28]. IL-1 KO mice that had been backcrossed for more than ten generations into the C57BL/6 strain were used in these experiments. Wild-type C57BL/6 mice were purchased from Charles River Laboratories (Tokyo, Japan). All mice were housed in the specific pathogen-free animal facility at Showa University and had free access to food and water. In all experiments, adult males 8 to 12 weeks old weighing 17 to 25 g were used. All experimental procedures involving animals were approved by the Institutional Animal Care and Use Committee of Showa University (#09156, 00136, 00139, and 01157).

### Spinal cord injury (SCI) model

The SCI mouse model was produced according to a previous report [29] with minor modifications. Anesthesia was induced in mice by inhalation of 4.0% sevoflurane and maintained with 3.0% sevoflurane. Under aseptic conditions, an incision was made along the midline of the skin of the back and the muscles, soft tissues and yellow ligaments overlying the spinal column between T9 and T10 were removed. The intervertebral spinal cord between T9 and T10 was then transected with a thin-bladed knife. After bleeding had stopped and coagulated blood was removed, the incision was closed and animals were given 1.0 mL physiological saline (s.c.) to avoid dehydration. Following recovery, foods were placed on the cage floor and the intake of the water bottle was lowered to allow for easy access. All mice were allowed to recover in a room maintained at 24 ± 1°C during the experimental period.

### Assessment of motor function

Motor function after SCI was compared by using an open field behavior test that focused on hindlimb function according to the Basso Mouse Scale (BMS) [30]. The BMS consists of an open-field locomotor rating scale, ranging from 0 (complete paralysis) to 9 (normal mobility). Briefly, individual mice were placed in the center of the open-field (for example, 50 × 50 cm square) with a smooth, non-slip floor and monitored for four minutes. Hindlimb movements, trunk/tail stability and forelimb-hindlimb coordination were assessed and graded. Mice were tested daily for 14 days post operatively (dpo). Mice with peritoneal infection, hindlimb wounds, and/or tail or foot autophagia were excluded from the study. Scoring was done by randomly numbering the mice to ensure that the investigators were not aware of the treatment groups.

### Measurement of injured area

After anesthesia with sodium pentobarbital (50 mg/kg, i.p.), animals were perfused transcardially at 3, 7 or 14 dpo with 0.9% saline followed by 10% buffered formalin and the spinal cord removed (T5 - L1 vertebrae). Spinal cords were then post-fixed and prepared in a paraffin block. Five spinal cord sections (5-μm thickness) were obtained from each mouse: at the midline which included the central canal near the core-injury site and bilaterally at 150 μm and 300 μm lateral to the midline (Total 5 sections from each mouse). The damaged area can be identified by glial fibrillary acidic protein (GFAP) immunostaining of the surrounding area which is considered to be indicative of glial scarring. The paraffin sections were deparaffinized and boiled in 10 mM citrate buffer (pH 6.0) at 90°C for 20 minutes. Following incubation in 0.3% H_2_O_2_, the sections were blocked with 5% normal horse serum (NHS) for 1 hour at room temperature. Subsequently, the sections were incubated overnight with rabbit anti-GFAP antibody (1:10, DAKO, Glostrup, Denmark). The sections were washed with PBS and immersed with goat anti-rabbit IgG (1:200, Santa Cruz Biotechnology, Santa Cruz, CA, USA) for 90 minutes. They were then incubated in an avidin-biotin complex solution (Vector, Burlingame, CA, USA) followed by diaminobenzidine (DAB; Vector) as a chromogen. Control staining involved carrying out the same steps minus the incubation with the primary antibody. The injured area surrounded by GFAP-immunopositive cells was measured by image analysis software DP2-BSW (Olympus, Tokyo, Japan), and the estimated lesion area was calculated by the average of the injury areas.

### Isolation of primary microglial cells from adult CNS

Isolation of primary adult microglia was carried out according to previous studies [31,32] using a slightly modified protocol. Briefly, anesthetized mice (sodium pentobarbital, 50 mg/kg, i.p., n = 20) were perfused transcardially with ice-cold saline under sterile conditions and the whole brain and upper spinal cord were quickly removed. The CNS tissues were homogenized with a Dounce-tissue grinder (Weaton, Millville, NJ, USA) and further digested by gentle shaking in a digestion cocktail (0.025 U/mL DNase (Sigma, St. Louis, MO, USA), 0.5% dispase II (Roche, Mannheim, Germany), 0.05% collagenase D (Roche), 0.1 μg/kg TLCK (Nα-Tosyl-L-lysine chloromethyl ketone hydrochloride; Sigma) in HBSS (Hank's balanced salt solution; Invitrogen, Carlsbad, CA, USA)). After filtration with a nylon filter (pore size 100-μm), the homogenate was centrifuged at 400 × g for six minutes and the pellet washed with HBSS and centrifuged again. Then, the pellet was resuspended in 30% isotonic Percoll (GE, Uppsala, Sweden), HBSS overlaid on the suspension, and the Percoll gradient solution was centrifuged at 200 × g for 40 minutes. After removing the debris and supernatant, the pellet was collected, washed, and resuspended with 10% RPMI1640 medium (10% heat-inactivated FCS (Nichirei, Tokyo, Japan), 2 mM L-glutamate, 100 units/ml penicillin and 100 μg/ml streptomycin in RPMI1640 (all from Invitrogen)) after HBSS washing. The cell suspension was seeded in six-well plates and incubated with a change of medium every three to four days for two weeks until confluency was reached (Additional file 1: Figure S1). The epitope profile of the cells was determined by immunocytostaining with antibodies against CD11b (AbD Serotec, Oxford, UK), Neu N (Millipore, Billerica, MA, USA, a neuronal marker), GFAP (DAKO, astroglial marker), and myelin basic protein (MBP, Millipore, an oligodendroglial marker).

### Activation of primary microglia with cytokines

MΦ can be activated into several kinds of polarized phenotypes depending on the stimulant [3]. We have reported that the BV-2 mouse microglial cell line can be activated according to the classical or alternatively activated phenotypes in response to stimulation by IFNγ or IL-4, respectively [33], with the phenotypes being similar to those described in previous reports [3]. Primary MG cultures produced from wild-type and IL-1 KO mice were washed twice with PBS (-) and replaced with experimental medium (D)MEM (Invitrogen) supplemented with 1% FCS, 100 U/ml penicillin, 100 μg/ml streptomycin, and 2 mM L-glutamine). Then the cells (n = 6 in each phenotype) were exposed to recombinant mouse IFNγ (rmIFNγ, 10 ng/mL), recombinant mouse IL-4 (rmIL-4, 20 ng/mL) or vehicle. Cells from half of the culture dishes were further exposed to 10 ng/mL rmIL-1β (all recombinant cytokines from PeproTech, Rocky Hill, NJ, USA). Twenty-four hours later, the medium and cells were collected and were kept at -30°C until analysis (Additional file 1: Figure S1).

To determine the contribution of IL-1β on alternative activation of MG, another set of primary MG were prepared as above. After culturing for two weeks, the MG were treated with rmIL-4 (20 ng/mL), rmIL-13 (20 ng/mL) or both (20 ng/mL each) with and without rmIL-1β. The cells and medium were collected 24 hours after treatment and were kept at -30°C until analysis (Additional file 2: Figure S2).

### Multiple-Immunostaining

Animals from the 3rd, 7th or 14th dpo were placed under sodium pentobarbital (50 mg/kg, i.p.) anesthesia and perfused transcardially with 0.9% saline followed by 4% paraformaldehyde (PFA) in 50 mM phosphate buffer (pH7.2). The T5 - L1 segment of the spinal cord was removed and tissues were post-fixed in fixative solution overnight, followed by 20% sucrose in 0.1 M phosphate buffer (pH7.2) for two nights. Tissues were then embedded in O.C.T. compound (Sakura Finetech, Tokyo, Japan) and frozen in liquid nitrogen-cooled isopentane. Ten-micron-thick sections were cut saggitally on a cryostat. Frozen sections of spinal cord from mice subjected to SCI were used for immunohistochemical staining. Primary cultures of microglia-rich cells were cultured in four- or eight-well permanox chamber slides (Nunc, Rochester, NY, USA), fixed with 2% PFA for 30 minutes, and used for immunocytostaining to determine the epitope profiles of the cells.

Tissue sections or chamber slides were washed several times with 0.1% Tween 20 in PBS (PBST) and incubated in 5% NHS/PBST for 1 hour. Subsequently, the sections were incubated overnight with primary antibodies. The sections were then rinsed with PBST and immersed with appropriate fluorescently-labeled secondary antibodies for 2 hours. Control staining involved carrying out the same procedures but without the incubation with primary antibodies. The primary and secondary antibodies used are listed in Tables 1 and 2. Some sections were stained with 4, 6-diamidine-2-phenylindole dihydrochloride (DAPI, 1:10,000; Roche) to identify cell nuclei. Fluorescence was detected using an Axio Imager optical sectioning microscope with ApoTome (Zeiss; Oberkochen, Germany).

**Table 1:** List of primary antibodies used for immunoblotting (IB), immunohistochemistry (IHC), and immunocytochemistry (ICC)

Primary antibodies	Clone #	Host	Company	Catalog #	Application	Folds
Arginase-1	19/Arginase I	Mouse	BD Pharmingen (Franklin Lakes, NJ)	61708	WB	2,000
β-Actin	AC-74	Mouse	Sigma (St Louise, MO)	A5316	WB	4,000
CD11b	5C6	Rat	Serotec (Oxford, UK)	MCA711	ICC	250
Cyclooxygenase 2 COX2)		Rabbit	Cayman Chemical (Ann Arbor, MI)	160106	WB	8,000
F4/80	CI:A3-1	Rat	BMA Biomedicals (Augst, Swizerland)	T-2008	IHC	500
GAPDH	6C5	Mouse	Chemicon International (Temecula, CA)	MAB374	WB	4000
Glial fibrially acidic protein (GFAP)	G-A-5	Mouse	Sigma (St Louise, MO)	G3893	ICC	250
		Rabbit	DAKO (Glostrup, Denmark)	N1506	IHC	10
Iba-1		Rabbit	WAKO (Osaka, Japan)	019-19741	IHC	500
Insulin-like growth factor 1	Sm1.2	Mouse	Upstate (Lake Placid, NY)	05-172	IHC	100
(IGF-1)						
Interleukin-1β (IL-1β)		Goat	R & D systems (Minneapolis, MN)	AF-401-NA	IHC	100
CD206	MR5D3	Rat	AbD Serotec (Raleigh, NC))	MCA2235GA	WB	4,000
myelin basic protein (MBP)		Rat	Chemicon International (Temecula, CA)	MAB386	ICC	250
NeuN		Mouse	Chemicon International (Temecula, CA)	MAB377	ICC	250
Neuron specific enolase (NSE)	3-(3)-C	Mouse	IBI (Fujioka, Japan)	11031S	IHC	1,000
Inducible nitric oxide synthase (iNOS/NOS type II)		Rabbit	Transduction Laboratories (Lexington, KY)	N32030	WB	10,000
STAT1	42	Mouse	Transduction Laboratories (Lexington, KY)	S21120	WB	5,000
Ym1		Rabbit	StemCell Tech (Vancouver, BC, Canada)	01404	WB	1,500

**Table 2:** List of secondary antibodies used for immunoblotting (IB), immunohistochemistry (IHC) and immunocytochemistry (ICC)

Secondary antibodies(conjugation)	Host	Company	Catalog #	Application	Folds
Mouse IgG (HRP)	Sheep	GE Healthcare Bioscience (Little Chalfont, UK)	NA931	WB	2,000
Rabbit IgG (HRP)	Donkey	GE Healthcare Bioscience (Little Chalfont, UK)	NA934	WB	4,000
Rat IgG (HRP)	Goat	GE Healthcare Bioscience (Little Chalfont, UK)	NA935	WB	5,000
Rabbit IgG (biotinylated)	Goat	Santa Cruz Biotechnology (Santa Cruz, CA)	SC-2040	IHC	200
Mouse IgG (Alexa 488 or 546)	Goat	Molecular Probes (Eugene, OR)	A11029 or A11030	IHC/ICC	400
Rabbit IgG (Alexa 488 or 546)	Goat	Molecular Probes (Eugene, OR)	A11034 or A11035	IHC	400
Goat IgG (Alexa 488)	Donkey	Molecular Probes (Eugene, OR)	A11055	IHC	400
Rat IgG (Alexa 488 or 546)	Goat	Molecular Probes (Eugene, OR)	A11006 or A11081	IHC/ICC	400

### Sample preparation and ELISA

Mice from the 1st, 3 rd, 7th and 14th dpo groups were placed under pentobarbital (50 mg/kg, i.p.) anesthesia and perfused with 0.9% NaCl, following which spinal cord segments between the T5 and L1 vertebrae were removed. The tissues were homogenized with lysis buffer (10 mM Tris-HCl (pH 7.4), 0.15 M NaCl and 1% Triton X-100, 1 mM ethylene glycol tetraacetic acid (EGTA), 50 mM NaF, 2 mM sodium orthovanadate, 10 mM sodium pyrvate, and protease inhibitor cocktail (Sigma)) and centrifuged at 800 × *g *for 10 minutes at 4°C, and the supernatant collected. Protein concentrations in the samples were determined using the BCA protein assay kit (Thermo Fisher Scientific, Waltham, MA, USA).

IL-1β, TNFα, and/or insulin-like growth factor 1 (IGF-1) protein levels were determined using a mouse IL-1β/IL-1 F2 kit (DY401), a mouse TNF-α/TNFSF1A kit (DY410) and a mouse IGF-1 kit (DY791) respectively, all of which were from R&D Systems (Minneapolis, MN, USA). Analyses were performed according to the manufacturer's instructions, and data were standardized according to total protein concentration.

For cell culture experiments, the culture media were spun down to remove cells and the supernatants used for the protein detection assays as described in the preceding paragraph. All *in vivo *and *in vitro *samples were stored at -30°C until use.

### Assay for arginase activity and NO production in primary cultures of MG

Arginase is a marker for alternative activation and its activity was measured according to our previous report [33]. Briefly, primary cultures of MG cells were sonicated with lysis buffer on ice. The homogenate was mixed with an equal volume of pre-warmed 50 mM Tris-HCl, pH 7.5 containing 10 mM MnCl_2 _and incubated for 15 minutes at 55°C for activation. The mixture was then incubated in 0.25 M L-arginine for 60 minutes at 37°C to hydrolyze urea from L-arginine, and the reactions were stopped by adding Stop solution (H_2_SO_4_/H_3_PO_4_/H_2_O, 1:3:7). Then, a 1% (final concentration) solution 1-phenyl-1,2-propanedione-2-oxime (ISPF, Wako, Tokyo, Japan) in ethanol was added to the solution, which was heated at 100°C for 45 minutes. The reaction between urea and ISPF produced a pink color, and absorption was measured at 540 nm. Data are presented as specific activity (nmol/min/mg of protein).

NO production is a marker for the classical activation of MΦ [3] and its level in cultured media was measured using the Griess method (Dojindo, Kumamoto, Japan) as NOx (NO_2_^-^and NO_3_^-^) according to the manufacturer's instructions.

### Western blot analysis

Immunoblotting experiments were carried out on spinal cord sections and cell homogenates. After determination of the protein concentration, the homogenates were prepared as reduced (except for CD206) or non-reduced (for CD206) immunoblotting samples. Then, appropriate amounts of samples were electrophoresed and ftransferred to polyvinylidinene fluoride membranes (Bio-Rad, Hercules, CA, USA). After blocking with 5% non-fat milk, the membranes were probed with primary antibodies for Ym1, STAT1, cyclooxygenase 2 (COX2), iNOS, arginase-1, CD206, and glyceraldehyde 3-phosphate dehydrogenase (GAPDH) or β-actin (as an internal control) overnight at 4°C. The membrane was rinsed with 10 mM Tris/HCl (pH 7.4) containing 0.05% Tween 20 (TBST) and probed with horseradish peroxidase (HRP)-conjugated secondary antibodies. Protein bands were detected by chemiluminescence (SuperSignal West Dura Extended Duration Substrate; Pierce, Rockford, IL, USA) and exposed onto X-ray film (Fuji film, Tokyo, Japan). The films were scanned and the signal densities were quantified using the UN-SCAN-IT gel analysis program (Silk Scientific, Orem, UT, USA). The densitometric data were corrected by an internal control and expressed as arbitrary units (unit). The primary and secondary antibodies used are listed in Tables 1 and 2.

### Statistical analysis

Each mouse was assigned a random number and all data were collected and analyzed without investigator knowledge of group identities. Data are expressed as mean ± SEM (standard error of the mean) for *in vivo *experiments and as mean ± SD for *in vitro *experiments. Statistical comparisons were made by Student's *t-*test for two groups and by one-way analysis of variance (ANOVA) followed by Dunnett's *post-hoc *tests for multiple groups. A value of *P *< 0.05 was considered statistically significant.

## Results

### SCI is suppressed in IL-1 KO mice

**Figure 1:**
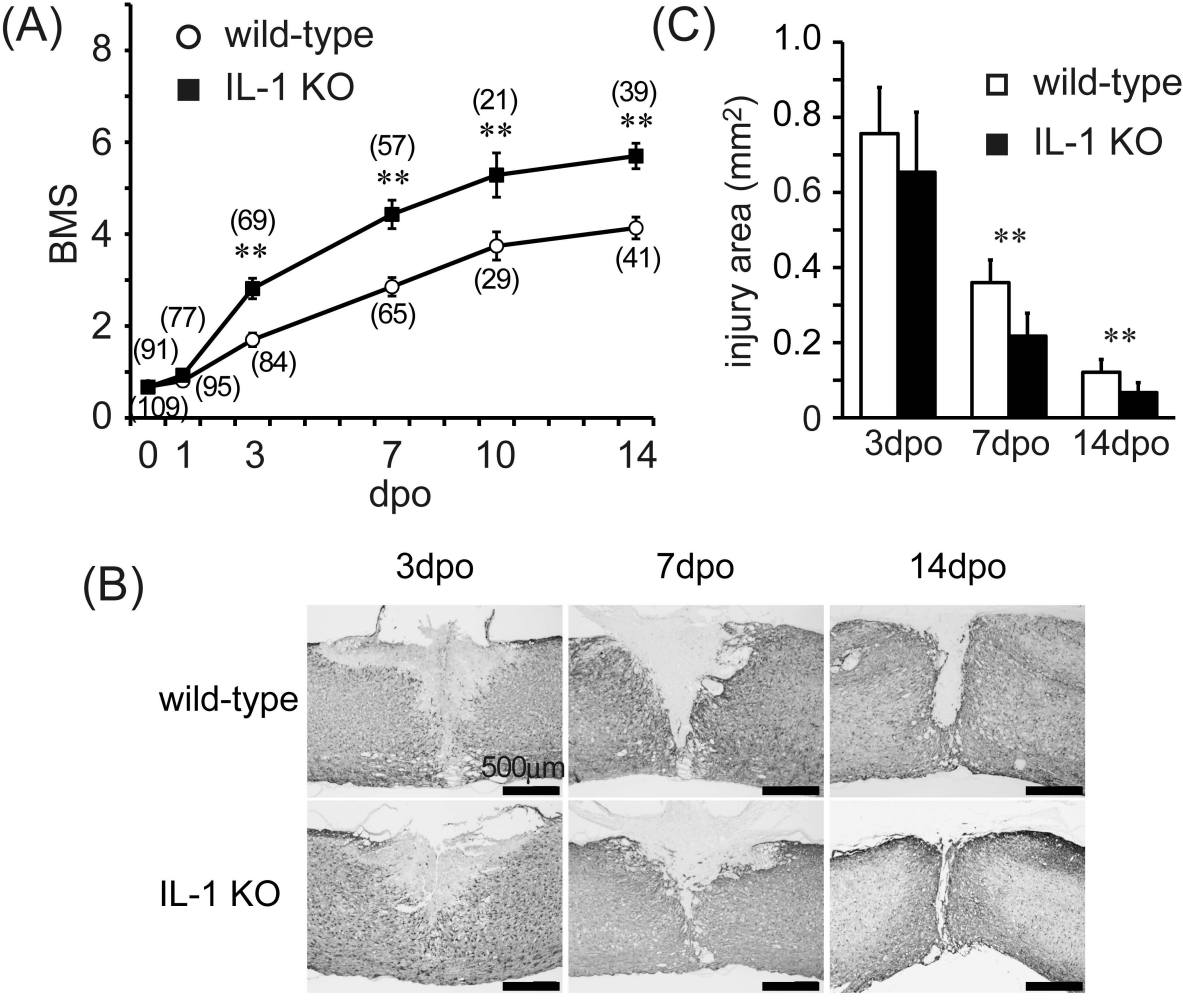
**SCI was suppressed more in IL-1 KO than in wild-type mice**. (**A**) Motor function determined according to the Basso Mouse Scale improved in a time-dependent manner after SCI in wild-type (open circles) and IL-1 KO mice (closed squares). The score for IL-1 KO mice is significantly improved compared to the wild-type from the 3rd dpo. Numbers in parentheses indicate sample numbers at each time point. Data are expressed as mean ± SE. **: *P <*0.01 (Student's t-test). (**B**) Representative images of GFAP-immunostaining after SCI. The injured area as defined by GFAP-positive cells is relatively smaller in IL-1 KO mice than in the wild-type. (**C**) The injured area is significantly suppressed in IL-1 KO mice (n = 5 to 7, filled bars) at the 7th and the 14th dpo compared to wild-type (n = 7 to 10, open column). Data are expressed as mean ± SE. **: *P <*0.01 (Student's t-test). dpo, days post-operatively; GFAP, glial fibrillary acidic protein; SCI, spinal cord injury.

Motor function determined by BMS declined quickly after SCI (0.67 ± 0.10 in each group) and then recovered gradually up to the 14th dpo in both the IL-1 KO mice and wild-type controls (Figure 1A). While there was no significant difference between BMS values on the 1st dpo, the scale levels improved significantly in IL-1 KO mice from the 3rd dpo (2.72 ± 0.20, *P < 0.05*) through the 14th dpo (5.60 ± 0.26, *P < 0.05*) compared with the day-matched wild-type animals (1.70 ± 0.14 and 4.13 ± 0.23, 3rd and 14th dpo, respectively).

The size of the injury area was compared between wild-type and IL-1 KO mice by using immunostaining to delineate the area surrounded by GFAP-positive cells (glial scar) (Figures 1B, C). The damaged site around the epicenter decreased through to the 14th dpo. The lesion area was calculated from the spatially-integrated injured area for both groups, with the lesion area in the IL-1 KO mice being significantly less than that in the wild-type animals; significant differences were measured at the 7th (0.21 ± 0.06 mm^2 ^IL-1KO versus 0.35 ± 0.06 mm^2 ^wild-type, *P <*0.05) and 14th dpo (0.06 ± 0.02 mm^2 ^IL-1KO versus 0.12 ± 0.03 mm^2 ^wild-type, *P <*0.05). These results indicate that IL-1 contributes to development of the lesion after SCI.

### IL-1 KO mice showed decreased induction of proinflammatory cytokines

**Figure 2:**
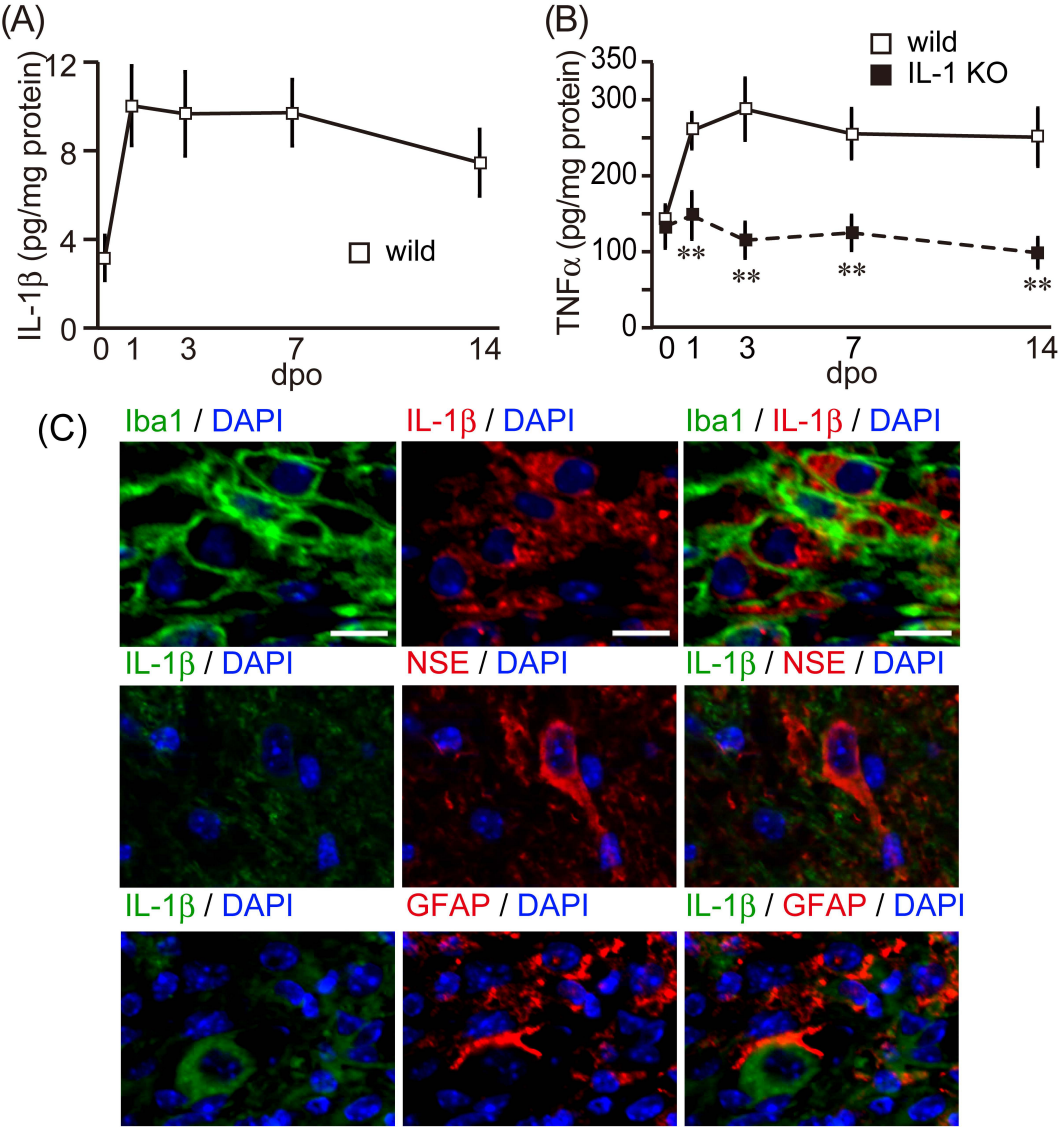
**Less proinflammatory cytokine induction was evident in IL-1 KO mice after SCI**. (**A**) IL-1β levels in the spinal cords of wild-type mice (open squares and solid line) were drastically increased from the 1st dpo and sustained through to the 14th dpo. No IL-1β was detected in the IL-1 KO mice (data not shown). (**B**) TNFα level in the spinal cord of the wild-type (open squares and solid line) and IL-1 KO mice (closed square and dotted line). TNFα expression in the wild type mice was increased from the 1st dpo and remained elevated through to the 14th dpo. However, TNFα levels in the IL-1 KO mice remained relatively unaltered post-operatively and were significantly lower than those in the wild-type mice. Data are expressed as mean ± SE. **: *P <*0.01 (Student's t-test). (**C**) Merged images of immunostaining for IL-1β and the microglia/macrophage marker, Iba-1. Minimal overlap of staining is observed between IL-1β, NSE and GFAP. Nuclei staining was detected with DAPI. DAPI, 4, 6-diamidine-2-phenylindole dihydrochloride; Dpo, days post-operatively; SCI, spinal cord injury.

IL-1β was barely detectable in the spinal cords of wild-type mice before SCI. The IL-1β level increased after SCI, peaked at 9.27 ± 1.79 pg/mg protein on the 1st dpo, remained high through to the 7th dpo, and then slightly decreased to 6.69 ± 1.49 pg/mg protein on the 14th dpo (Figure 2A). No IL-1β signal could be detected in IL-1 KO mice (data not shown). We examined another proinflammatory cytokine, TNFα, the level of which also increased after SCI (Figure 2B). TNFα in the wild-type animals peaked at 289.0 ± 41.0 pg/mg protein on the 3rd dpo and remained high through to the 14th dpo. There was no significant difference in the TNFα level between groups prior to the SCI. However, the TNFα level in the IL-1 KO mice was significantly lower than that in the wild-type mice from the 3rd dpo (*P <*0.01) and remained constant during the experimental period. IL-1β-positive cells were identified using multiple-immunostaining at three days (Figure 2C). Immunoreactivity for IL-1β was detected well at the epicenter of lesion site. The positive cells were co-localized with Iba1-expressing cells as an MΦ/MG marker. However, an astroglial marker, GFAP-positive reactions were observed around the lesion site. A neural marker, NSE-positive cells, decreased in the lesion epicenter and also in the peri-lesion and was observed in the outside. Therefore, GFAP- and NSE-positive cells were hard to co-observe with IL-1β-positive cells (Figure 2C). IL-1β was not expressed in the IL-1 KO mice (data not shown).

### IL-1 KO mice show decreased alternative activation of microglia

**Figure 3:**
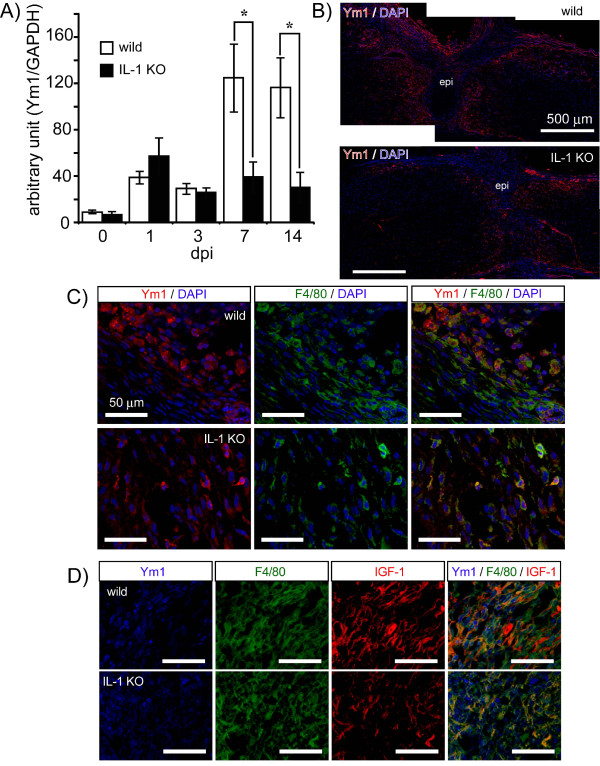
**IL-1 KO mice do not exhibit increased expression of the alternative activation marker, Ym1, after SCI**. (**A**) Expression of the marker for alternatively activated microglia/macrophage, Ym1, was semi-quantified by immunoblotting. Ym1 levels in the wild-type mice (n = 6 to 7, open column) were increased on the 7th and 14th dpo. However, the level of Ym1 in the IL-1KO mice (n = 5 to 6, filled column) is significantly lower than that for the wild-type mice. Data are expressed as mean ± SE. *: *P <*0.05 (Student's t-test). (**B**) Immunostaining at the 14th dpo for Ym1 in the spinal cord after injury. The immunoreactivity of Ym1 (red) is observed around the lesion epicenter (epi) and is more intense in the wild-type than in IL-1 KO mice. (**C**) Ym1 immunoreactivity (red) merges with F4/80 immunoreactivity (green, microglia/macrophage marker) in both groups of animals. (**D**) Moreover, Ym1 (blue) co-localizes with cells that are immunopositive for F4/80 (green) and IGF-1 (red) in both groups. Dpo, days post-operatively; SCI, spinal cord injury.

Alternative activation of MΦ/MG gene expression increases in the sub-acute stage after SCI [34]. Our results were consistent with this, as we found that levels of Ym1 protein, a marker of alternatively activated MΦ/MG, were slightly upregulated post-operatively. Compared with its level on the 1st dpo, Ym1 expression increased approximately three-fold in wild-type mice in the subacute phase on the 7th and 14th dpo after SCI (Figure 3A). The level of Ym1 expression in the IL-1 KO mice was similar to that of the wild-type mice on the 1st dpo; however, in contrast to the wild-type mice there was no increase observed on the 7th and 14th dpo, and levels remained significantly lower. Ym1-immunopositive cells were identified using multiple-staining and found to be present mainly around the lesion site (Figure 3B). There was no obvious difference in the distribution of these cells between the IL-1 KO mice and their wild-type counterparts; however, the density of the positively labeled cells was greater in the wild-type mice. Merged images showing immunoreactivity for Ym1 and F4/80 (a MΦ/MG marker) in both the wild-type and IL-1 KO mice (Figure 3C) and for F4/80 and IGF-1 (Figure 3D) demonstrated that these markers were co-expressed in the same cells in both groups, although to a lesser extent in the IL-1 KO mice. These results suggest that the absence of IL-1 might result in a lower level of MΦ/MG alternative activation.

### MG activation is influenced by IL-1β

To confirm the contribution of IL-1 to increase Ym1 and to promote the alternative activation of MΦ/MG, we established primary cultures of MG from adult mouse CNS tissue and stimulated these cells by exposing them to either IFNγ or IL-4 with and without IL-1β.

**Figure 4:**
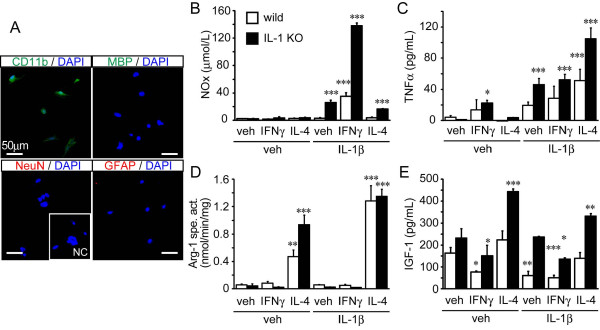
**Activation of adult primary microglial cells in wild-type and IL-1 KO mice**. (**A**) Primary microglial cells were obtained from young adult wild-type mice. The cells stain with the microglial marker CD11b, but not with the neuronal and astroglial markers, NeuN and GFAP, respectively. A few cells are stained with the oligodendroglial cell marker, MBP. NC (inset) is the primary antibody-free negative control. The microglial cells (n = 3 each group) were stimulated for 24 hours in the presence of the vehicle alone, or supplemented with IFNγ or IL-4 in the presence or absence of IL-1β. Total NO (NOx; **B**), TNFα (**C**), arginase specific activity (Arg-1 spe. act.; **D**) and IGF-1 (**E**) were determined from the media or cell suspensions. (**B**) NOx levels increase upon exposure of the cells to IL-1β and in a synergistic manner upon co-treatment of cells with IL-1β and IFNγ, but not when the cotreatment is with IL-4. (**C**) TNFα levels increase upon exposure of the cells to IFNγ, and further upon co-treatment with IL-1β. Surprisingly, the co-treatment of the cells with IL-4 and IL-1β induced the highest TNFα level among the experimental treatments used. (**D**) Arg1-specific activity increased significantly upon exposure to IL-4 and further increased when IL-4 and IL-1β were employed together. (**E**) IGF-1 levels decreased with exposure of the cells to IFNγ and increased in response to IL-4. The response was partially inhibited by cotreatment of the cells with IL-1β. Data are expressed as mean ± SD (n = 3). *: *P *< 0.05, **: *P *< 0.01, ***: *P *< 0.001 compared with the vehicle-treated group in each genotype (one-way ANOVA followed by Dunnett *post-hoc *test). ANOVA, analysis of variance; IGF-1, insulin-like growth factor.

We first determined the epitope characteristics of the MG by immunocytostaining with markers for microglial (CD11b), neural (MAP2), astroglial (GFAP) and oligodendroglial (MBP) cells (Figure 4A). Most of the cultured cells stained positively for CD11b antibody, while none of them stained with GFAP or NeuN antibodies. We observed that a few cells were stained by the oligodendroglial marker MBP and estimated that > 95% of the cells were MG.

We then examined the effect of rmIFNγ (10 ng/mL) and rmIL-4 (20 ng/mL) on the microglial activation (Figures 4B-E). IFNγ is an inducer of the classical activating phenotype while IL-4 is an inducer of the alternative activating phenotype [3]. In the presence of these cytokines, we first measured IL-1β levels in the media after a 24-hour incubation to determine the level of endogenous IL-1β. However, no endogenous IL-1β was detected by ELISA (data not shown). Minimal levels of NOx measured by Griess methods were detected in the standard MG culture media and this did not change in the presence of IFNγ or IL-4 (Figure 4B). The level of TNFα increased significantly 24 hours later in IFNγ-treated cultures of MG cells from both wild-type and IL-1 KO mice compared with untreated cultures, but not in response to IL-4 treatment (Figure 4C). The degree of activation was much more evident when the cells were exposed to a combination of IFNγ and IL-1β. Exposure of cultured MG from IL-1 KO mice to IL-1β alone increased the level of NOx in the culture media, while co-treatment with IFNγ increased NOx even further in a synergistic manner. However, co-treatment of cultured MG from the IL-1 KO mice with IL-4 and IL-1β also the increased NOx level, although to a lesser extent than that seen with the IFNγ and IL-1β co-treatment (Figure 4B). TNFα also increased in response to IL-1β treatment alone, but not synergistically for the IFNγ and IL-1β co-treatment. Surprisingly, although treatment of IL-4 alone in MG cells did not increase TNFα in the media, co-treatment with IL-4 and IL-1β did result in an increase of TNFα in the media (Figure 4C).

In contrast, treatment of IL-4 alone in MG cells significantly increased arginase-1 activity in both wild-type and IL-1 KO mice. Co-treatment of IL-4 with IL-1β gave rise to a synergistic increase of arginase-1 activity, while treatment with IL-1 alone did not (Figure 4D). IGF-1 levels increased in response to exposure to IL-4 and decreased in the presence of IFNγ. Co-treatment of IL-4 with IL-1β in MG showed a similar effect to that seen following treatment of IL-4 alone, although most of all experimental groups tended to decrease with the addition IL-1 treatment (Figure 4E).

**Figure 5:**
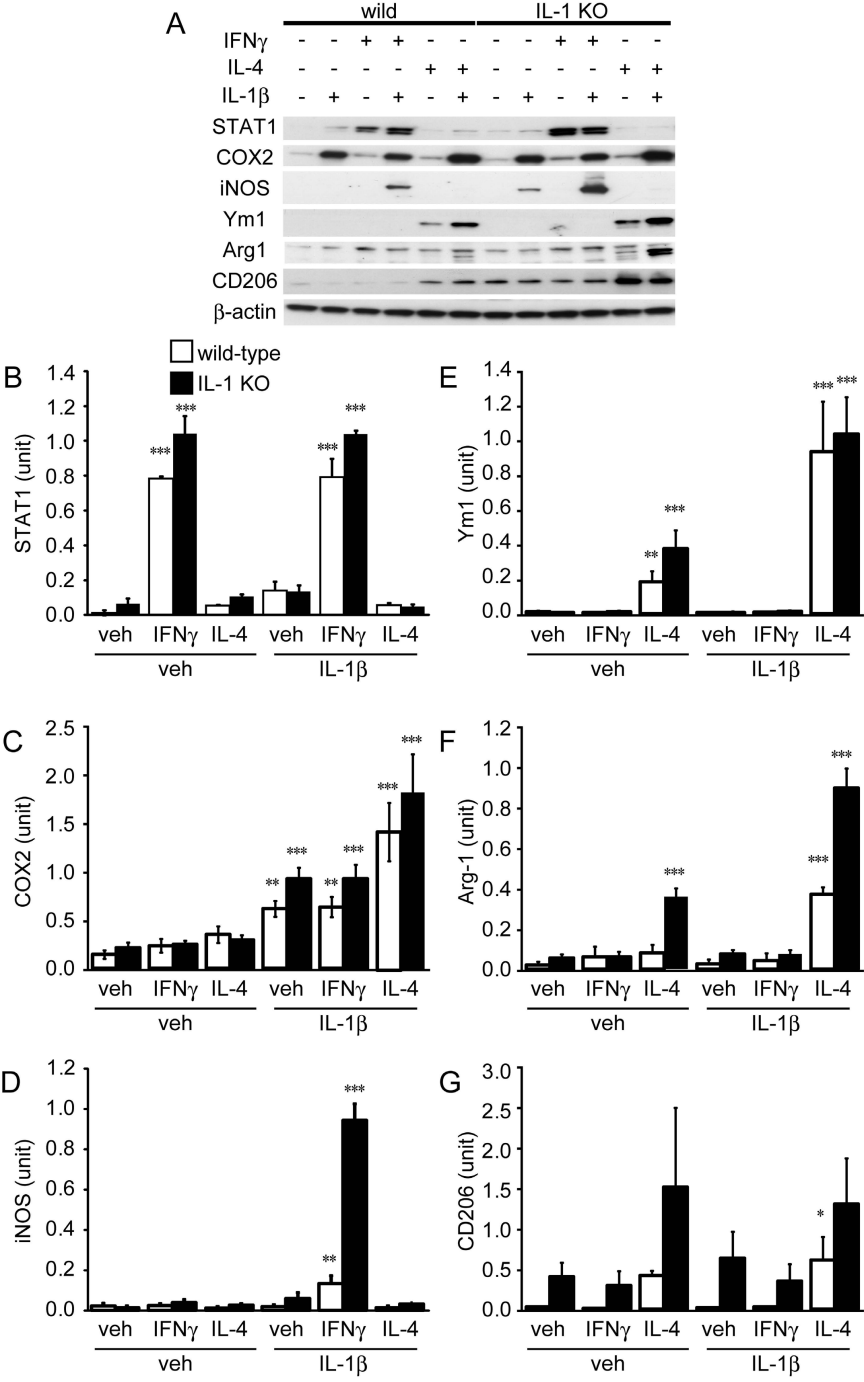
**Western blot analysis to identify the type of activation of adult primary microglial cells from wild-type and IL-1 KO mice stimulated by vehicle, IFNγ or IL-4 with or without IL-1β**. (**A**) Representative western blotting data of primary MG produced from wild-type (wild) and IL-1 KO mice exposed for 24 hours to vehicle, IFNγ or IL-4 with or without IL-1β. Each lane expected to CD206 blotting applied 8 μg of reduced samples. Non-reduced samples (5 μg) were applied to detect CD206. Densitometric analysis of STAT1 (**B**), COX2 (**C**), iNOS (**D**), Ym1 (**E**), Arg-1 (**F**) and CD206 (**G**) (n = 3 each group). (B) STAT1 level is increased by exposure of cells to IFNγ, but not to IL-4. The level is not influenced by co-treatment of cells with IL-1β. (C) The COX2 level is increased by exposure of cells to IL-1β but not to IFNγ or IL-4 alone. Co-treatment with IL-1β and IL-4 tends to increase the COX2 level synergistically. (D) While the iNOS level is not increased significantly by exposure of cells to IFNγ or IL-4, the level increased synergistically in response to co-treatment with IFNγ and IL-1β. (D) The level of Ym1 is increased by exposure of cells to IL-4 and further increased by co-treatment with IL-1β. (E) The Arg-1 level is also increased by exposure of cells to IL-4 and further increased by co-treatment with IL-1β. (G) The CD206 level is increased slightly by exposure of cells to IL-4, but not with co-treatment with IL-4 and IL-1β. Data are expressed as mean ± SD (n = 3). *: *P *< 0.05, **: *P *< 0.01, ***: *P *< 0.001 compared to the vehicle-treated group without IL-1β for each genotype (One-way ANOVA followed by Dunnett *post-hoc *test). ANOVA, analysis of variance; arg-1, arginase 1; COX2, cyclooxygenase 2; iNOS, inducible NO synthase.

Further characterization of the form of MG activation was carried out with immunoblotting experiments (Figure 5). STAT1 was mostly upregulated by IFNγ and IL-4 alone or co-treatment IL-4 with IL-1β did not influence the signals (Figure 5A, B). COX2 was mostly upregulated by IL-1β. While IFNγ did not influence the signals, co-treatment IL-4 with IL-1β tended to increase the signals synergistically (Figure 5A, C). iNOS expression evolved in a similar manner to that of NOx production (Figure 5A, D). Its signal could not be detected in response to the incubation of cultured MG cells with IFNγ or IL-4 alone. Co-treatment of the cells with IFNγ and IL-1β, however, resulted in a significant increase of iNOS levels. Levels of Ym1 and arginase-1, as markers of alternative activation, increased similarly with arginase-1 activity. Ym1 and arginase levels increased in response to treatment with IL-4 alone (Figure 5A, E, D), but significantly more so in response to co-treatment with IL-4 and IL-1β. Another marker of alternative activation, CD206 (mannose receptor), also increased in response to treatment of cells with IL-4 in the presence/absence of IL-1β (Figure 5A, G).

### MG are polarized to the alternative activating phenotype by IL-4 and not by IL-13

**Figure 6:**
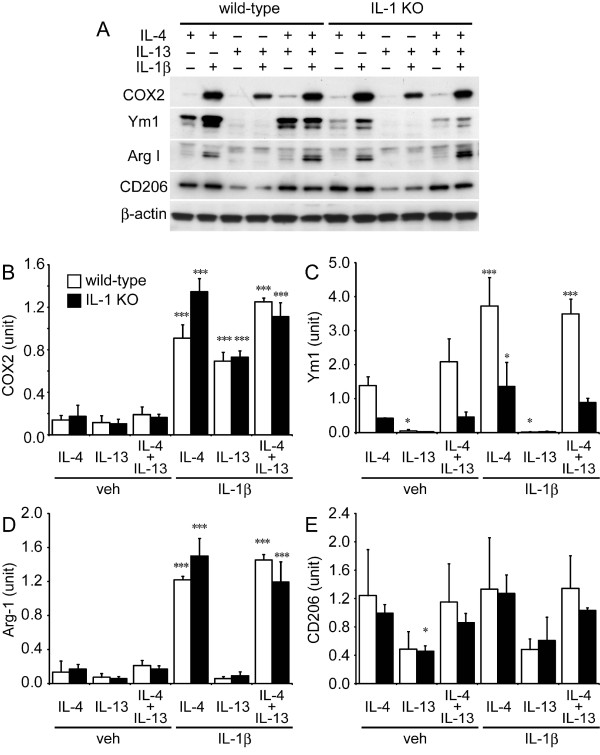
**Western blot analysis to determine type of activation of adult primary microglial cells produced from wild-type and IL-1 KO mice stimulated with IL-4, IL-13 or IL-4/IL-13 with or without IL-1β**. (A) Representative western blotting data of primary microglial cells produced from wild-type (wild) and IL-1 KO mice and exposed for 24 hours to IL-4, IL-13 or IL-4 plus IL-13 (IL-4/IL-13) with or without IL-1β. Each lane expected to CD206 blotting were applied 7 μg of reduced samples. Non-reduced samples (5 μg) were applied to detect CD206. Densitometric analysis of COX2 (B), Ym1 (C), Arg-1 (D) and CD206 (E) (n = 3 each group). (B) COX2 levels are increased by exposure of cells to IL-1β and are not influenced by IL-4 or IL-13 alone. The COX2 level was slightly enhanced by IL-1β and IL-4 co-treatment. (C) Ym1 levels are increased by exposure of cells to IL-4 and IL-4/Il-13 and are synergistically increased by co-treatment with IL-1β. However, only a low level of Ym1 is seen upon exposure of cells to IL-13, and is significantly less than that seen in response to exposure of cells to IL-4. (D) Arg-1 shows similar levels in response to exposure to IL-4 and IL-4/IL-13; these are synergistically increased by co-treatment with IL-1β. However, low levels of Arg-1 are seen for exposure of cells to IL-13. (G) CD206 was detected in response to exposure of cells to both IL-4 and IL-4/IL-13 with or without IL-1β; however, CD206 levels in IL-13-exposed samples were lower than those seen with the other treatments. Data are expressed as mean ± SD (n = 3). *: P < 0.05, **: P < 0.01, ***: P < 0.001 compared with the IL-4-treated group without IL-1β for each genotype (one-way ANOVA followed by Dunnett post-hoc test). ANOVA, analysis of variance; arg-1, arginase 1; COX 2, cyclooxygenase 2.

It is known that the alternative activation of MΦ induced by IL-4 and IL-13 is mediated by the IL-4 receptor pathway [3,5,35]. As such, we treated MG cell cultures with rmIL-4 (20 ng/mL), rmIL-13 (ng/mL) or IL-4 plus IL-13 (IL-4/IL-13) in the presence or absence of IL-1β (Figure 6). IL-1β-treated cell lysates showed similarly increased COX2 levels to that seen in experiments described in Figure 5C (Figure 6A, B), while IL-4-treated MG again showed increased Ym1 expression. However, in the presence of IL-13, MG cell lysates did not show any increase in Ym1, nor when IL-1β was added exogenously to the culture medium. IL-4/IL-13 treatment showed a mostly similar expression of Ym1 to that seen with IL-4 treatment alone for MG cells from both the wild-type and IL-1 KO mice (Figure 6A, C). Arginase-1 levels increased dramatically and in a similar manner in response to exposure of cells to IL-4 alone and for IL-4/IL-13 co-treatment with IL-1β. Arginase-1 levels either did not change, or only changed slightly in response to exposure to IL-13 alone and were not enhanced by IL-13 and IL-1β co-treatment (Figure 6A, D). CD206 signals from MG cells were similar in response to IL-4 or IL-4/IL-13 treatment, and did not change in response to additional IL-1β co-treatment. However, cells treated with IL-13 in the presence or absence of IL-1β showed CD206 signals that were approximately half that seen for the IL-4 and IL-4/IL-13 treatments (Figure 6A, E). These results suggest that cultured adult MG cells take on the alternative activation phenotype in response to IL-4 and are less likely to do so in the presence of IL-13. Again, the alternative activation of the MG is modulated by additional IL-1β.

## Discussion

MΦ/MG have a diverse range of functions during CNS diseases depending on the type of induction caused by unique cytokine stimuli. Although many papers have reported that the MΦ/MG play a role in the induction of inflammation and neural cell death by releasing pro-inflammatory cytokines and producing oxidative stress, recent evidence also suggests the a phenotype of MΦ/MG contributes to the repair and regenerative process after the diseases [3-5]. In the present study, we demonstrated the MΦ/MG activating phenotypes 14 days after SCI. Moreover, to observe the influence of IL-1, we compared the lesion size and MΦ/MG activation using IL-1 KO mice. Our studies clearly showed that IL-1 KO mice have a smaller lesion size and less motor deficit than the wild-type mice. Interestingly, although IL-1 KO mice had a suppressed TNFα level, an inflammatory marker, from the 1st dpo, the animals also had a decreased Ym1 level which is an alternative activating MΦ/MG marker at the 7th and 14th dpo. To confirm the phenomenon, we established adult mouse primary MG cultures, and examined cell responses to the cytokines IFNγ and IL-4 directly with and without IL-1β. These results suggest that IL-1 might participate in the classical and alternative activation of MΦ/MG.

Previous reports have suggested a contribution of IL-1 in acute CNS diseases such as SCI [13,14,16,36], cerebral ischemia [19-21], trauma [37,38], and subarachnoid hemorrhage [39]. However, no direct evidence from IL-1 KO mice has demonstrated the contribution of IL-1 to SCI. Our results were consistent with previous studies that IL-1 or IL-1 receptor signaling pathway contributes to increase lesion size of the SCI. An increase in IL-1β and a decrease in IL-1ra were observed after SCI, and IL-1β administered into the spinal cord impaired locomotion. Moreover, administering IL-1ra into the spinal cord reduced IL-1β levels and locomotion recovered [16]. IL-1 consists of two molecular subtypes, IL-1α and IL-1β [40]. IL-1α is expressed continuously while IL-1β is inducible in response to injury. We used IL-1α and β KO mice because it has been reported that IL-1α -or β-alone KO mice do not give rise to the neuroprotective phenotype after ischemia [20]. It has also previously been shown that a post-traumatic neuroinflammatory response was involved in the development of injury, and that IL-1 worked as a key inflammatory player that mediated the neuroinflammatory response [41]. This is supported by results showing that IL-1 recruits monocytes to enhance the inflammation mediated by IL-1 receptor I and via a MyD88-dependent pathway [42]. Down-regulation of the IL-1 receptor pathway and IL-1-mediated inflammatory responses becomes a strategy for the suppression of SCI. To this extent, several recent studies have shown that IL-1 receptor antagonists are able to reduce the severity of symptoms after experimental SCI [14,36,43,44]. From these results, we confirmed that our experimental condition did not differ obviously from the previous one and that a deficiency of IL-1 worked as a suppressor of SCI.

We then undertook experiments to determine whether IL-1 deletion affected the inflammatory response. Time-dependent changes in the levels of the pro-inflammatory cytokines IL-1β and TNFα in the spine after SCI were measured with ELISA. It has been reported that pro-inflammatory cytokines including IL-1β, IL-6, and TNFα are induced rapidly following SCI [45-49]. In the present study, IL-1β levels in the spine of wild-type mice after SCI were drastically increased from the 1st dpo and were sustained until the 14th dpo. Immunohistochemical studies suggested that IL-1β was expressed in MΦ and/or MG. The results suggested that IL-1 contributes to the inflammatory responses after SCI. The TNFα level in the wild-type mice was increased in the spine from the 1st dpo and was sustained until the 14th dpo. However, the TNFα level in IL-1 KO mice did not increase after injury and remained at significantly lower levels during the experimental period compared with that seen in wild-type mice. The results suggest that IL-1 participates in the upregulation of expression of TNFα, probably following the induction of a series of inflammation events after injury.

Recently, it has been suggested that IL-1β triggers the proliferation and early differentiation of neural progenitor cells during development of the spinal cord and after hippocampal injury [23,24]. Moreover, other inflammatory factors such as TNFα and iNOS were implicated in aspects of neural regeneration during wound-repair [26,27]. In the present study, we found that IL-1β levels remained high up to the 14th dpo despite a decrease in the size of the lesion site. We then postulated that IL-1β might be performing in a different role during part of this period, and, therefore, carried out immunoblotting experiments to examine Ym1 levels in response to SCI. Ym1 has been reported as an excellent marker of alternative activation of MΦ and/or MG [50], which is one of the activation phenotypes induced by IL-4 and IL-13 [3-5] and plays an important role in the resolution of inflammation and promotion of wound healing [3,50]. Alternative activating MΦ/MG gene expression increases during the sub-acute stage after SCI [34]. The alternative activation of MΦ promotes axonal growth and overcomes inhibitory substrates [8]. MΦ implanted into the injured spinal cord increase axonal regrowth and/or functional improvement [9,10,51,52]. Immunoblotting for Ym1 revealed higher levels at the 7th and 14th dpo in wild-type mice than in IL-1 KO mice, with immunoreactivity concentrated around the lesion epicenter in injured spinal cord. The Ym1 immunoreactivity coincided with that of immunoreactivity for F4/80 and the growth factor IGF-1, which is known to increase alternative activation of MΦ/MG and plays an important role in neuroprotection [11,53,54]. We postulated that IL-1 might contribute to Ym1 expression, and to the induction of alternative activation. Taken together, these results suggest that IL-1 increases the inflammatory response and might also increase tissue repair and anti-inflammatory resolution via the induction of alternative activation of MΦ/MG in response to SCI. Unfortunately, we were unable to differentiate between MG and MΦ because there is no specific immunohistochemical marker available to separate them.

Then, we established adult mouse primary MG cultures and examined cell responses to the cytokines IFNγ and IL-4. Moreover, we added IL-1β to this system to observe its effect because we could not detect endogenous IL-1β in the media after exposing cells to either IFNγ or IL-4 alone. We have previously reported that NOx and TNFα levels in the media of primary cultures of the mouse MG BV-2 line increased in response to exposure to IFNγ alone [33]. Other studies using rodent primary MG obtained from the pups and the BV-2 cell line have also shown an increased expression of inflammatory mediators (TNFα, IL-1β, IL-6, COX-2) and iNOS after MG stimulation by IFNγ and LPS [55-57]. In the present study, while the level of TNFα increased in response to IFNγ treatment, NOx did not. However, NOx was drastically increased by co-treatment with IFNγ and IL-1β; iNOS levels as determined by immunoblotting behaved similarly. Moreover, other alternative activation markers such as arg 1 (activity and protein level), IGF-1, Ym1 and CD206 [3,7,54] did not increase upon exposure to IFNγ in the presence or absence of IL-1β. These results indicate that MG polarizes to the classical activating phenotype by IFNγ and/or IL-1β [3,53]. Some minor differences with other studies exist, with discrepancies perhaps due to differences in the source and type of cells and experimental conditions used.

By contrast, MG exposed to IL-4 showed an increase of arginase activity, as well as increased arg-1, IGF-1, Ym1 and CD206 protein levels, but not NOx, iNOS or TNFα. These characteristics clearly indicated that the MG polarized to the alternative activating phenotype [3,53]. Surprisingly, co-treatment of MG with IL-4 and IL-1β further increased arg-1 activity, and arg-1 and Ym1 protein levels towards the alternatively activated phenotype. Because treatment of MG with IL-1β alone did not increase these factors, it is suggested that IL-1β has a supportive effect on IL-4-induced responses and supports the induction of the alternative activating phenotype in adult mouse MG. However, another alternative factor, CD206 was not enhanced and IGF-1 tended to decrease following IL-1β co-treatment with IL-4. The co-treatment of MG with IL-4 and IL-1β gave rise to an unexpectedly high TNFα level as well. Because exposure of MG to IL-4 alone did not increase the level of TNFα, the co-treatment is considered to be the result of a synergistic effect between IL-1β and IL-4. To date, no evidence has been reported to show that IL-4 works as an enhancer of the IL-1β response. IL-4/IL-13 has basically been considered to antagonize the IL-1β function [58] by enhancing the production of IL-1ra and the decoy IL-1β type II receptor [59,60]. Moreover, IL-4/IL-13 downregulated the pro-IL-1β cleavage enzyme, caspase 1, to convert it to an active mature form [61,62]. However, a small number of papers have reported that an alternative activating phenotype is classified into sub-phenotypes. A sub-phenotype of MΦ, M2b is influenced by IL-1β. It has been reported that M2b induces TNFα and IL-10 production [54,63,64]. However, the main role and phenotype of M2b remain unclear. Moreover, there is no evidence to show that IL-4 participates in the polarization of this phenotype. Different reactions of alternative markers by co-treatment of IL-4 and IL-1β might be due to sub-phenotypes of alternative activating MG. Further studies are needed to clarify the relation between the cytokine network and MG polarization.

Finally, we determined the possible involvement of IL-4 and IL-13 in the adult MG alternative activating response. Many research and review articles have indicated that both IL-4 and IL-13 function similarly as activators of alternatively polarized MΦ [7,35]; however this has not been studied in detail in adult MG. We applied IL-4, IL-13, or IL-4/IL-13 to primary cultures of adult MG with and without IL-1β to demonstrate a putative signaling mechanism for MG alternative activation, and found that Ym1, arg-1 and CD206 were enhanced by IL-4 and IL-4/IL-13, but not by IL-13 alone. Because the levels of induction between IL-4 and IL-4/IL-13 were very similar, we thought that the effect of induction depended on IL-4. Moreover, even if MG cultures were co-treated with IL-13 and IL-1β, the Ym1 and arg-1 did not further increase in the same manner as for IL-4. Two IL-4 receptors, type I IL-4 receptor (IL-4RI) and type II IL-4 receptor (IL-4RII), mediate IL-4's functions [7,35]. IL-4RI is exclusive for IL-4, while IL-4RII binds both IL-4 and IL-13. IL-4RI is expressed predominantly in hematopoietic lineage cells and IL-4RII is expressed in hematopoietic and non-hematopoietic cells [35]. Although we did not determine the expression of the IL-4 receptors, the present results suggest that adult MG are polarized to the alternatively activated phenotype by IL-4 but not by IL-13, and that some MG functions might be mediated through IL-4RI. Further analyses are required to determine what IL-4 receptor(s) is involved in the present phenotypes, and what differences exist between MG and MΦ in this respect.

Alternatively activated MΦ are now regarded as a continuum of functionally and phenotypically related cells, with a critical role in the resolution and tissue repair phases [3,7,54]. Indeed, it has been reported that immune cells contribute to the maintenance of neurogenesis and spatial learning abilities in adulthood because immune-deficient mice showed impaired hippocampal neurogenesis that could not be enhanced by environmental enrichment [65]. Previously, it was suggested that IL-1β itself contributed to the proliferation and differentiation of neural progenitor cells in the spine and hippocampus, and to nerve regeneration by promoting neurite outgrowth following nerve injury [23,24,66]. A mixture of murine recombinant IL-1β, IL-6 and TNFα administered to the lesioned spinal cord four days after the lesion significantly decreased the amount of tissue loss seven days after trauma compared with vehicle-administered controls [41]. Moreover, gene-deficient mice have been used to show that TNFα and iNOS are implicated in neural regeneration during wound-repair stages [26,27]. This accumulated evidence lends itself to the suggestion that the relationship between IL-1 and IL-4 and the alternative activation of MG might be implicated in neurogenesis. The manner in which the enhancement of alternative activation markers following co-treatment with IL-4 and IL-1β contributes to wound healing, repair and neurogenesis needs to be examined more in detail, as does the way in which immune/inflammatory responses tune the switching to resolution and regeneration following SCI and in other CNS diseases.

In conclusion, we have demonstrated here in *in vivo *experiments that IL-1 exacerbates the effects of SCI by accentuating the impact of the inflammatory responses. Moreover, the results of *in vivo *and *in vitro *experiments suggest that IL-1 participates in the classical and alternative activation of MG. Finally, we suggest that the alternative activation of adult MG is regulated via an IL-4 signaling pathway that could be mediated by IL-4RI.

## Abbreviations

ANOVA: analysis of variance; arg-1: arginase-1; BMS: Basso Mouse Scale; CNS: central nervous system; COX2: cyclooxygenase 2; DAPI: 4: 6-diamidine-2-phenylindole dihydrochloride; dpo: days post operation; EGTA: ethylene glycol tetraacetic acid; GAPDH: glyceraldehyde 3-phosphate dehydrogenase; GFAP: glial fibrillary acidic protein; hMSCs: human stem/progenitor cells (is also human marrow stroma cells or human mesenchymal stem cells); HRP: horseradish peroxidase; iNOS: inducible nitric oxide synthase; IFNγ: interferon-γ; IGF-1: insulin-like growth factor-1; IL-1: interleukin-1; IL-4: interleukin-4; IL-4R: interleukin-4 receptor; IL-6: interleukin-6; IL-13: interleuin-13; ISPF: 1-phenyl-1,2-propanedione-2-oxime; MG: microglia; MΦ: macrophage; NC: negative control; NHS: normal horse serum; NO: nitric oxide; NSE: neuron specific enolase; PFA: paraformaldehyde; rm: recombinant mouse; SCI: spinal cord injury; TNFα: tumor necrosis factor α.

## Competing interests

The authors declare that they have no competing interests.

## Authors' contributions

SA performed the animal experiments and data analysis, and prepared the initial version of the manuscript. HO performed the culture experiments using primary mouse microglial cells and prepared the manuscript. TT were substantial contributions to animal experiments. DS contributed to the isolation and characterization of primary mouse microglial cells. KO were substantial contributions to western blotting assay and immunohistochemistry. MA and YI provided IL-1 knockout mice. TA and SS supervised all experimental procedures. All of the authors have read and approved the final version of the manuscripts.

## Supplementary Material

Additional file 1 **Figure S1**. Diagram of primary cultural microglial study (1). Microglial cells were isolated from 20 adult mouse brains and upper spinal cords by Percoll density gradient. The cell suspension was plated on 3 of 6-well multiple plate. After culturing with RPMI based media for 2 to 3 weeks, the media were replaced by experimental medium and added the medium (as a vehicle), or IFNγ (10 ng/mL) or IL-4 (20 ng/mL) in the medium. Within a few minutes, IL-1β (10 ng/mL) was also added to half of the wells. The details of the methods are shown in Materials and Methods section. Click here for file

Additional file 2 **Figure S2**. Diagram of primary cultural microglial study (2). Microglial cells were isolated from 20 adult mouse brains and upper spinal cords by Percoll density gradient. The cell suspension was plated on 3 of 6-well multiple plate. After culturing with RPMI based media for 2 to 3 weeks, the media were replaced by experimental medium and added IL-4 (20 ng/mL), IL-13 (20 ng/mL), or IL-4 + IL-13 (20 ng/mL each) in the medium. Within a few minutes, IL-1β (10 ng/mL) was also added to half of the wells. The details of the methods are shown in Materials and Methods section. Click here for file
