# Supplementary figures and images for: Interleukin-1 participates in the classical and alternative activation of microglia/macrophages after spinal cord injury
Source: J Neuroinflammation. 2012 Apr 7;9:65. doi: 10.1186/1742-2094-9-65 (PMC3353190; doi:10.1186/1742-2094-9-65)

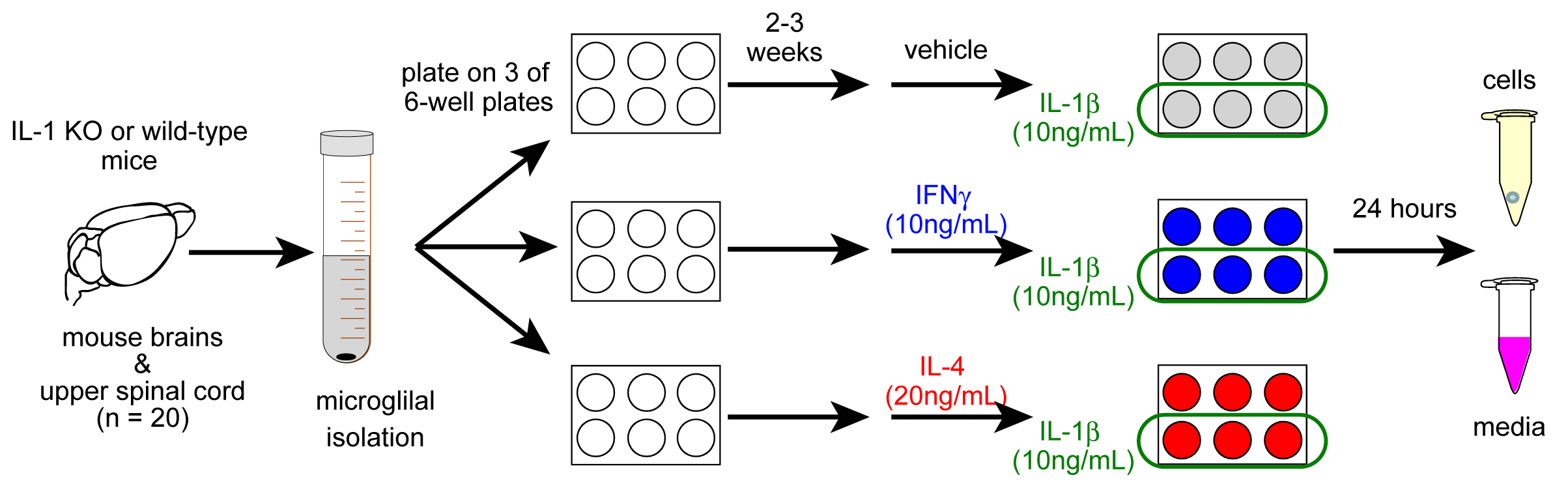

Supplement: Additional file 1 — Figure S1. Diagram of primary cultural microglial study (1). Microglial cells were isolated from 20 adult mouse brains and upper spinal cords by Percoll density gradient. The cell suspension was plated on 3 of 6-well multiple plate. After culturing with RPMI based media for 2 to 3 weeks, the media were replaced by experimental medium and added the medium (as a vehicle), or IFNγ (10 ng/mL) or IL-4 (20 ng/mL) in the medium. Within a few minutes, IL-1β (10 ng/mL) was also added to half of the wells. The details of the methods are shown in Materials and Methods section. [file 1742-2094-9-65-S1.TIFF]

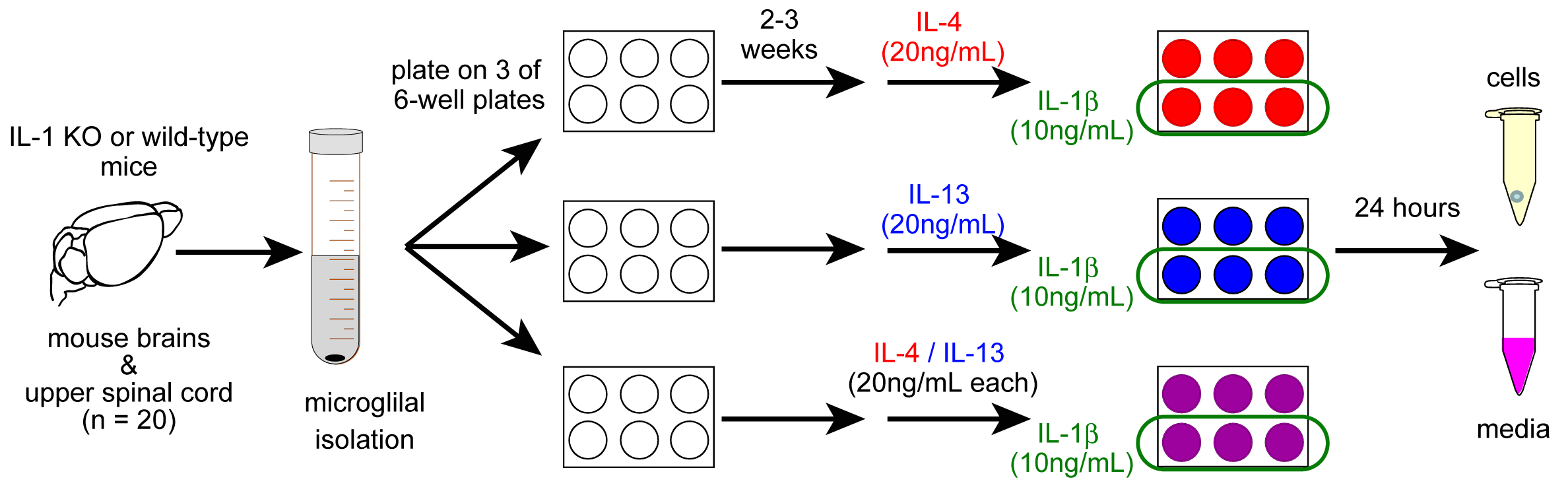

Supplement: Additional file 2 — Figure S2. Diagram of primary cultural microglial study (2). Microglial cells were isolated from 20 adult mouse brains and upper spinal cords by Percoll density gradient. The cell suspension was plated on 3 of 6-well multiple plate. After culturing with RPMI based media for 2 to 3 weeks, the media were replaced by experimental medium and added IL-4 (20 ng/mL), IL-13 (20 ng/mL), or IL-4 + IL-13 (20 ng/mL each) in the medium. Within a few minutes, IL-1β (10 ng/mL) was also added to half of the wells. The details of the methods are shown in Materials and Methods section. [file 1742-2094-9-65-S2.TIFF]
